# Supplementary figures and images for: Vinculin is required for interkinetic nuclear migration (INM) and cell cycle progression
Source: J Cell Biol. 2023 Oct 27;223(1):e202106169. doi: 10.1083/jcb.202106169 (PMC10609122; doi:10.1083/jcb.202106169)

Fig 2D

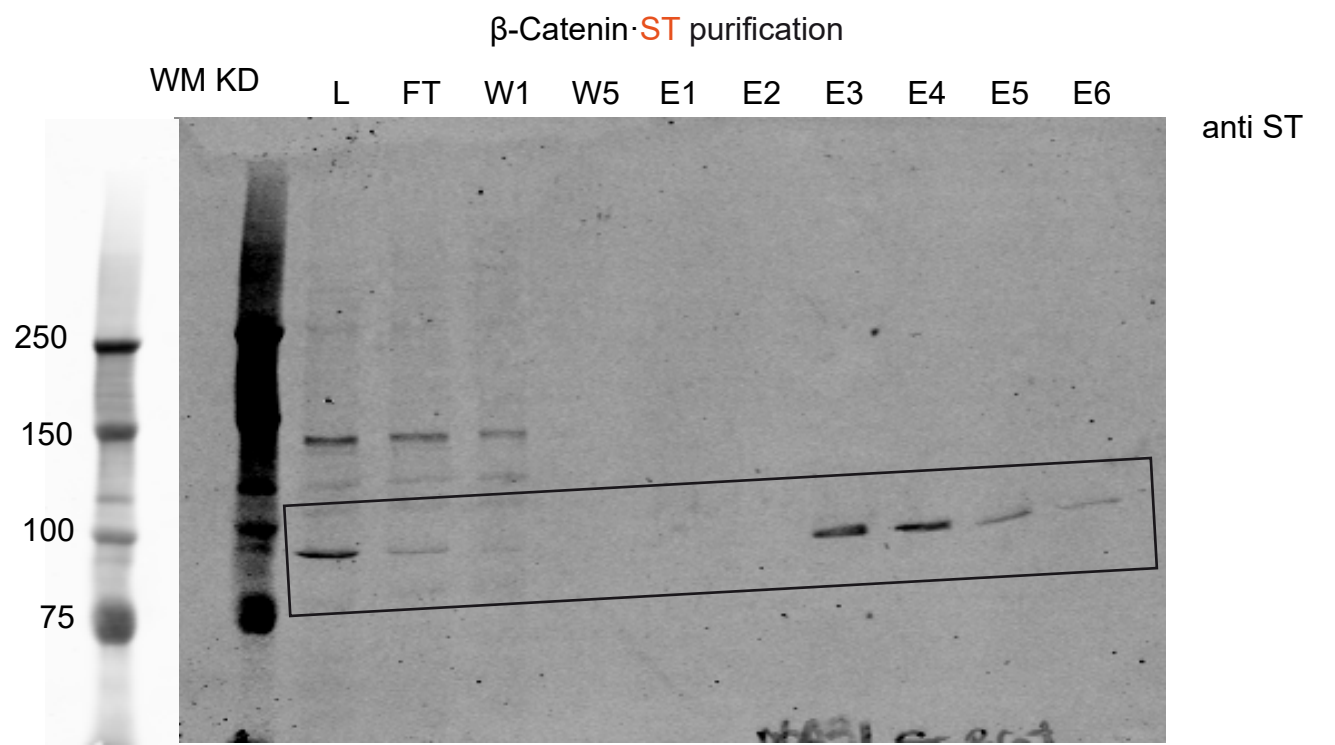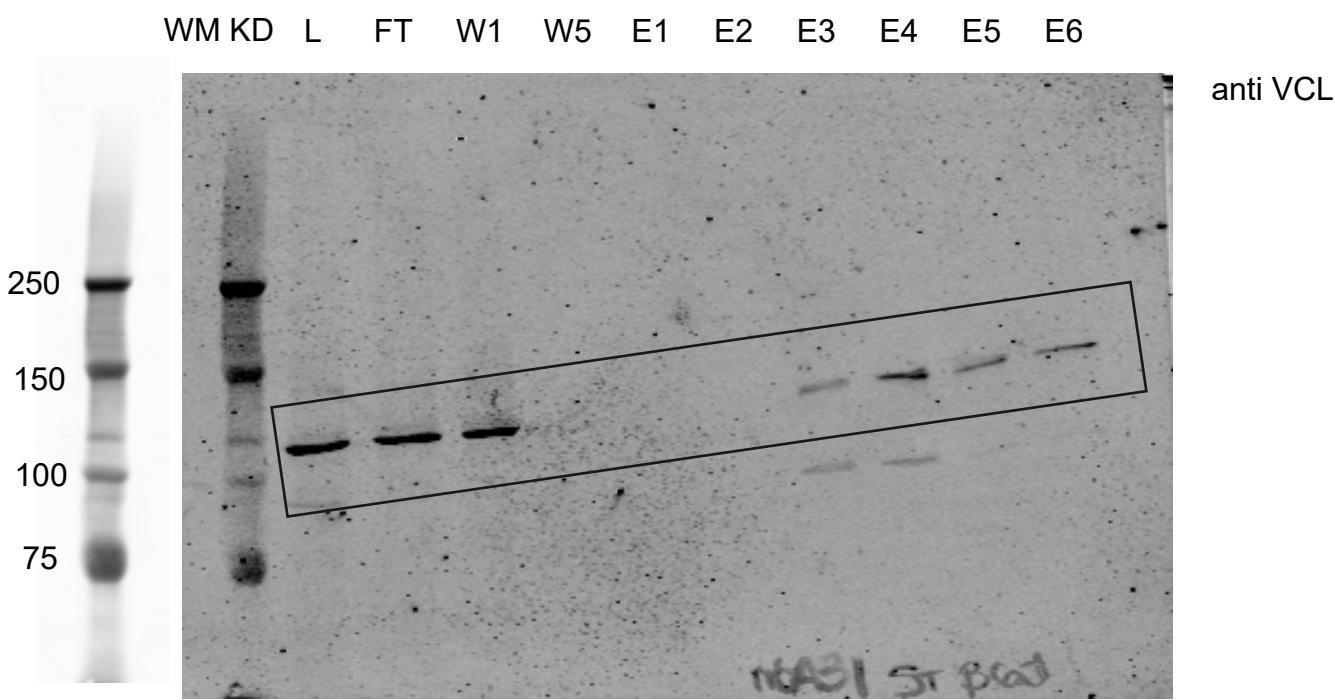

Supplement: SourceData F2 — is the source file for Fig. 2. [file JCB_202106169_SourceDataF2.pdf]
